# Supplementary figures and images for: Hormonal signaling cascades required for phototaxis switch in wandering Leptinotarsa decemlineata larvae
Source: PLoS Genet. 2019 Jan 7;15(1):e1007423. doi: 10.1371/journal.pgen.1007423 (PMC6336328; doi:10.1371/journal.pgen.1007423)

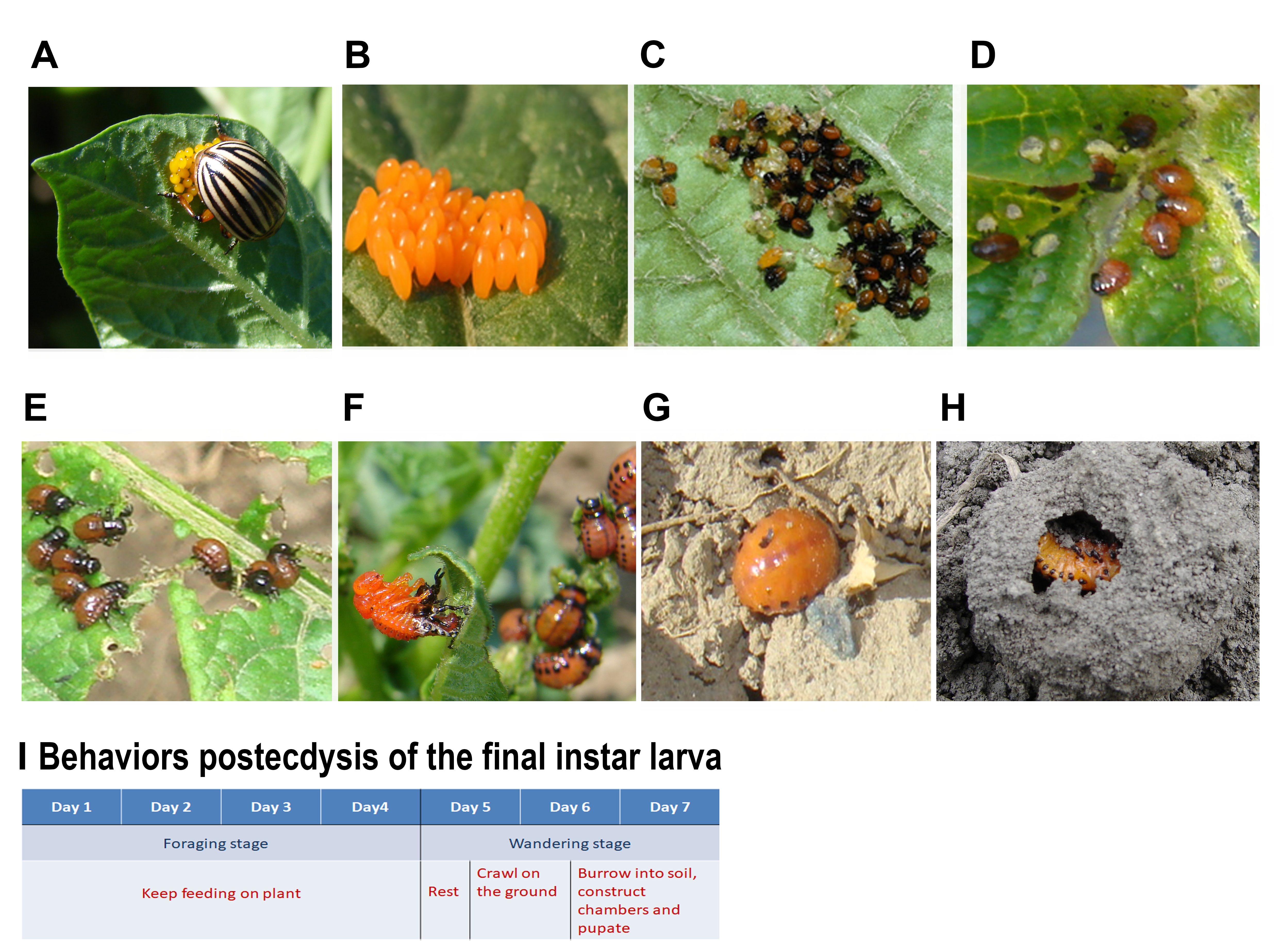

Supplement: S1 Fig — A total of 100 Leptinotarsa egg masses were selected randomly along a diagonal line. The location sites of egg masses (A and B), hatchlings (C), second- and third-instar larvae (D, E), fourth-instar larvae and a molting fourth-instar larva (F), wandering larvae (G) and prepupae (H) were observed. The duration from ecdysis to pupation of the final instar larvae lasts around 7.0 days (I). (TIF) [file pgen.1007423.s001.tif]

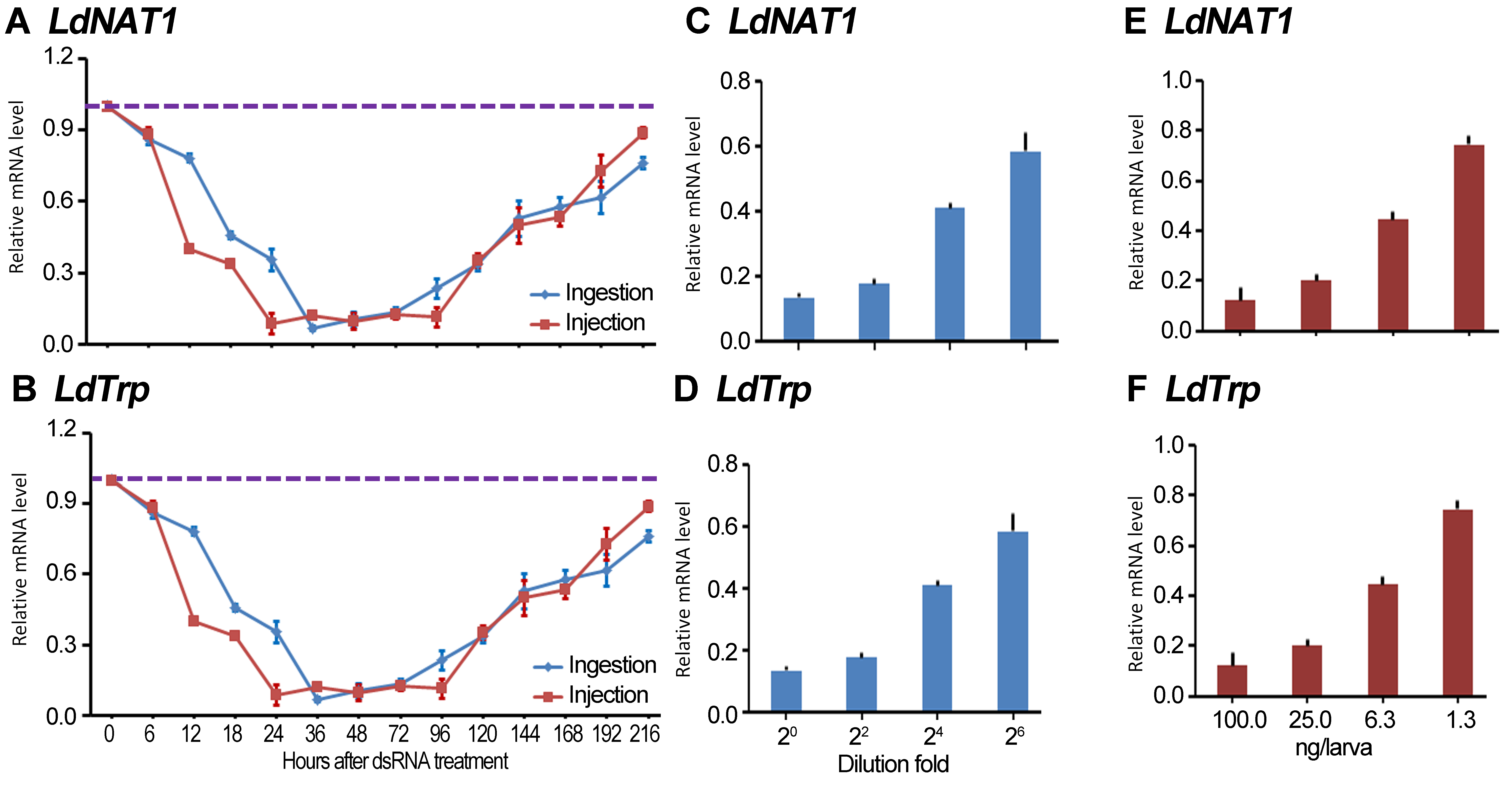

Supplement: S2 Fig — For time-effect curve, newly-ecdysed Leptinotarsa fourth-instar larvae had ingested foliage immersed dsNAT1- or dsTrp-contained bacterial solution for 3 days, or injected 50 ng of dsNAT1 or dsTrp into hemolymph. The relative levels of either LdNAT1 or LdTrp were measured (those in dsegfp-treated larvae were set as 1) at specific hours after experiment (A, B). For concentration-effect curve using dsRNA ingestion method, dsRNA-contained bacterial solution was diluted 0, 4, 16 and 64 folds with PBS, and used to immerse foliage. Newly-ecdysed Leptinotarsa fourth-instar larvae were allowed to ingest the treated-foliage for 36 hours (C, D). For concentration-effect curve using dsRNA injection method, Newly-ecdysed Leptinotarsa fourth-instar larvae were injected 100.0, 25.0, 6.3 and 1.3 ng of dsRNA, and test the relative expression levels 24 hours after injection (E, F). Larvae treated with dsegfp were controls whose expression levels of target genes were set as 1. (TIF) [file pgen.1007423.s002.tif]

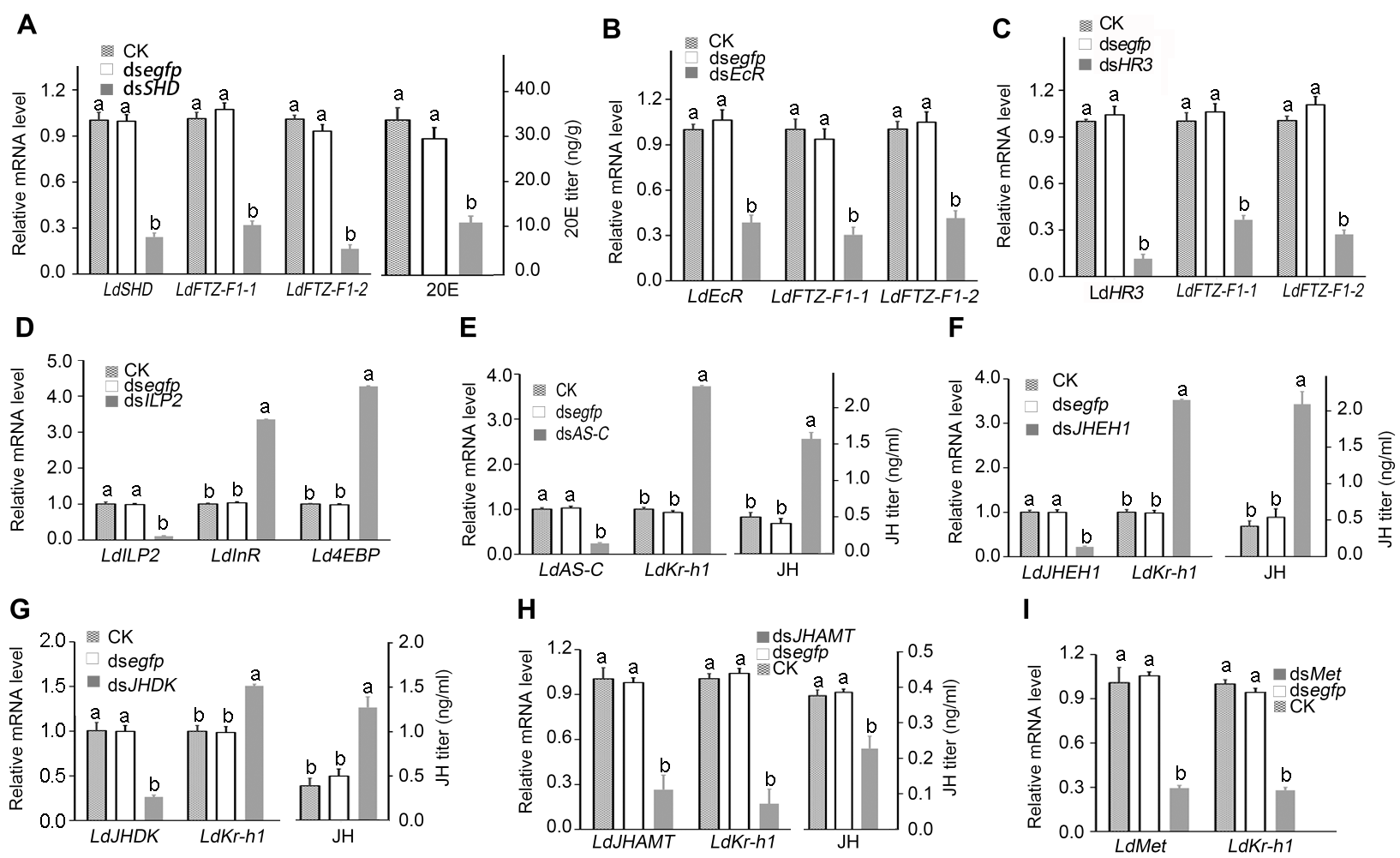

Supplement: S3 Fig — Newly-ecdysed Leptinotarsa third-instar larvae had fed on dsSHD-, dsEcR-, dsHR3-immersed foliage for 3 days. Newly-ecdysed Leptinotarsa fourth-instar larvae had ingested dsILP2-, dsAS-C-, dsJHEH1-, dsJHDK-, dsJHAMT- or dsMet-immersed foliage for 3 days. The larvae having fed PBS- or dsegfp-dipped foliage were set as controls. Expression levels were measured after the larvae having fed on dsRNA for three days. Significantly different mRNA levels (2-ΔΔCt values±SE, the ratios of copy numbers in treated individuals relative to those in blank controls) of target and a down-stream 20E signaling gene (LdFTZ-F1) (A, B, C), two down-stream insulin signaling genes (LdInR and Ld4EBP) (D), or a down-stream JH signaling gene (LdKr-h1) (E-I), and/or 20E (A) or JH (E-H) titers were marked with different letters (P<0.05). (TIF) [file pgen.1007423.s003.tif]

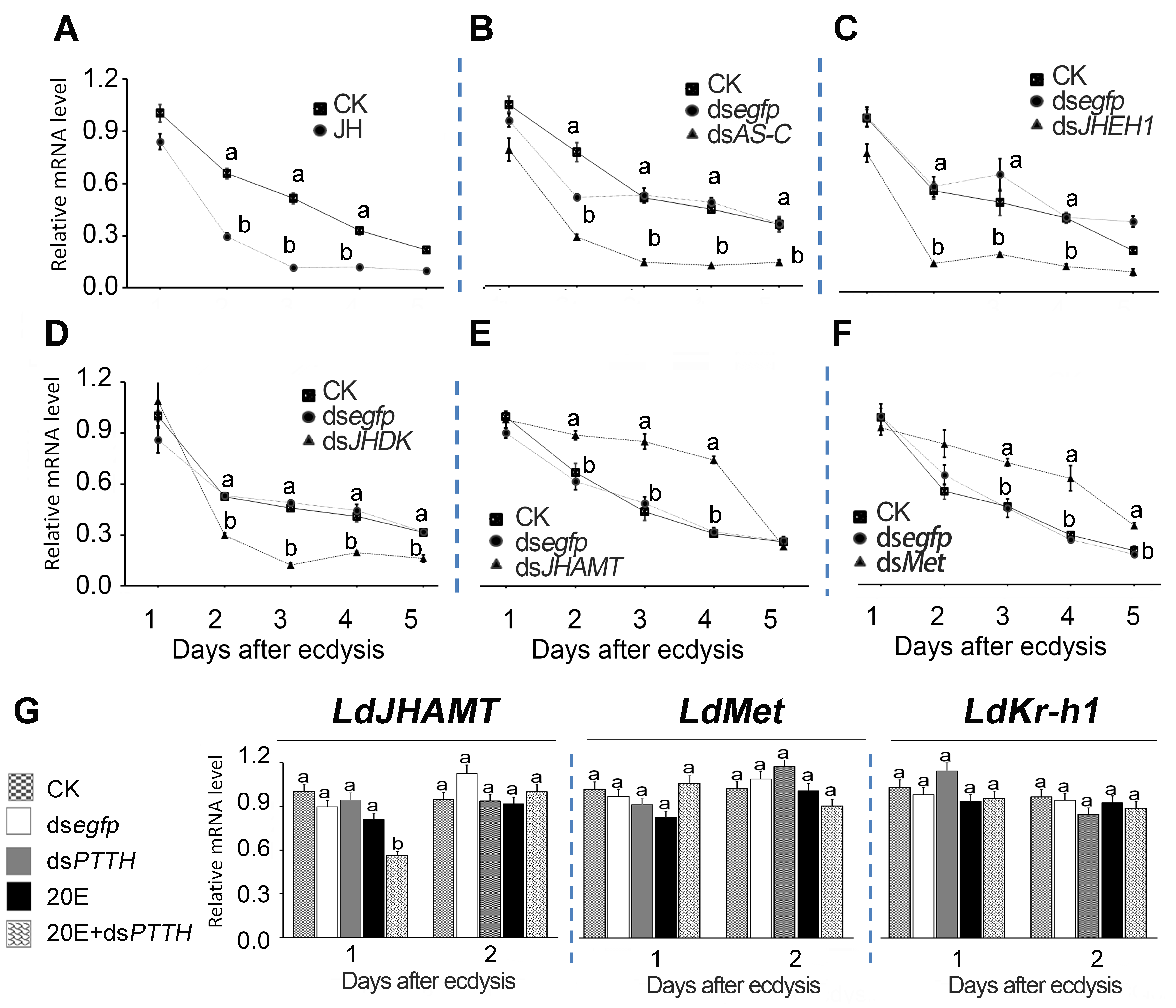

Supplement: S4 Fig — (A-F) Disturbance of JH signals influences the expression level of LdPTTH. JH signals were enhanced by JH ingestion (A), or knockdown of AS-C, JHEH1 and JHDK (B-D), whereas the signals were repressed by silencing of JHAMT and Met (E, F). The expression levels of LdPTTH were determined. (G) Knockdown of PTTH did not affect the expression levels of JH signal genes. The mRNA levels of LdJHAMT, LdMet and LdKr-h1 were tested at day 1 and 2 post ecdysis of the fourth-instar larvae. (TIF) [file pgen.1007423.s004.tif]

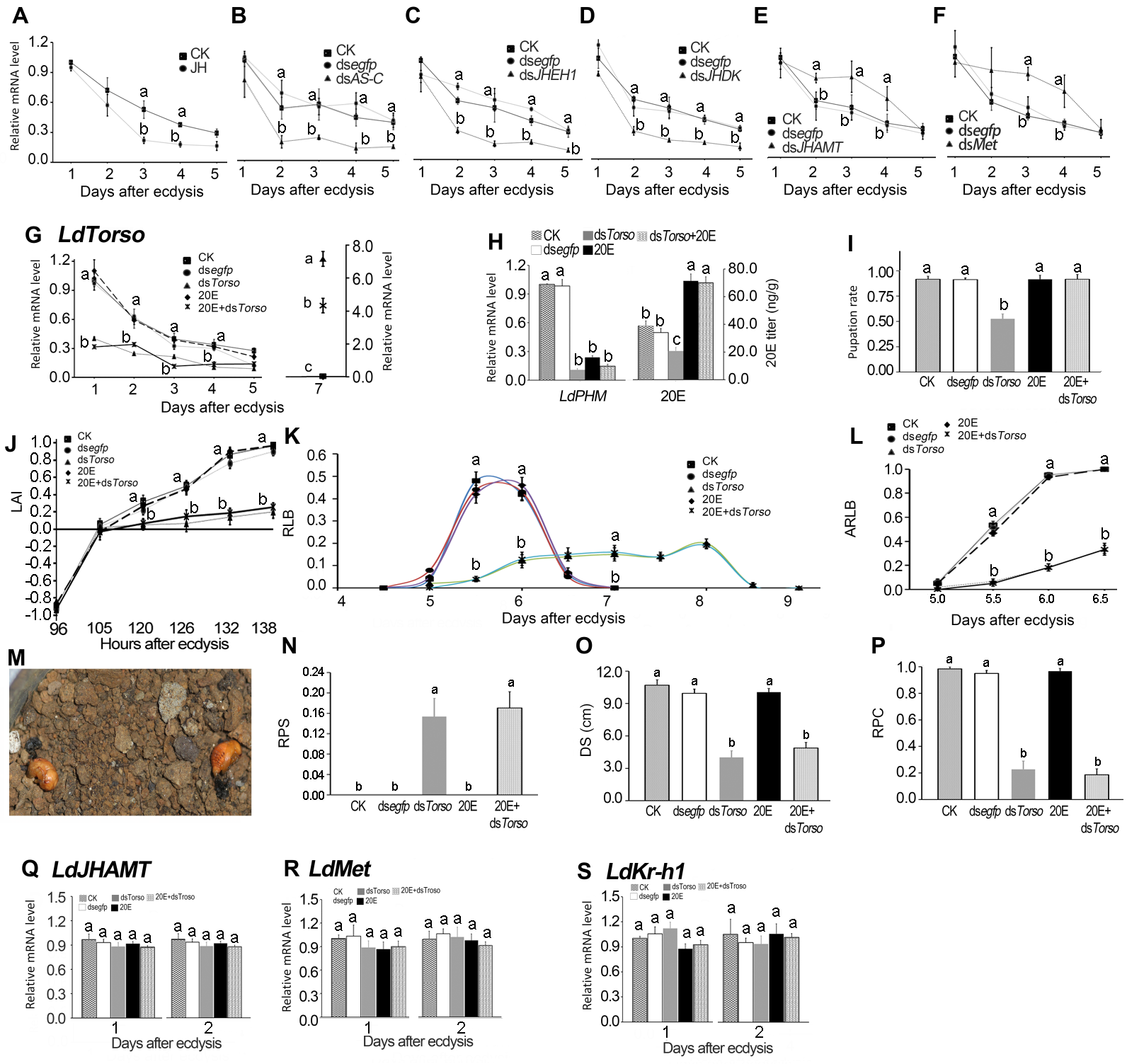

Supplement: S5 Fig — (A-F) Disturbance of JH signals influences the expression level of LdTorso. JH signals were enhanced by JH ingestion (A), or knockdown of AS-C, JHEH1 and JHDK (B-D), whereas the signals were repressed by silencing of JHAMT and Met (E, F). The expression levels of LdTorso were determined. (G-P) PTTH signal promotes light avoidance of the wandering larvae. PBS (CK), dsegfp, dsTorso, 20E and dsTorso+20E were dietarily introduced to the larvae. LdTorso mRNA level (G), light avoidance index (LAI) (J), rate of larvae that had buried in soil per day (RLB) (K), and accumulated RBP (ARLB) (L) were determined at each testing time point demonstrating in figure. LdPHM mRNA level and 20E titer (H) were tested at day 1 of the fourth-instar larvae. Pupation rate (I), rate of pupae at the soil surface (RPS) (M, N), average depths from pupation site to soil surface (DS) (O), and rate of pupae that had constructed pupation chambers (RPC) (P) were measured at the end of the experiment (P<0.05). Significant differences between blank control larvae (CK) and treatments are indicated by different letters (P<0.05). (Q-S) Knockdown of Torso did not affect the expression levels of JH signal genes. The mRNA levels of LdJHAMT, LdMet and LdKr-h1 were tested at day 1 and 2 post ecdysis of the fourth-instar larvae. (TIF) [file pgen.1007423.s005.tif]

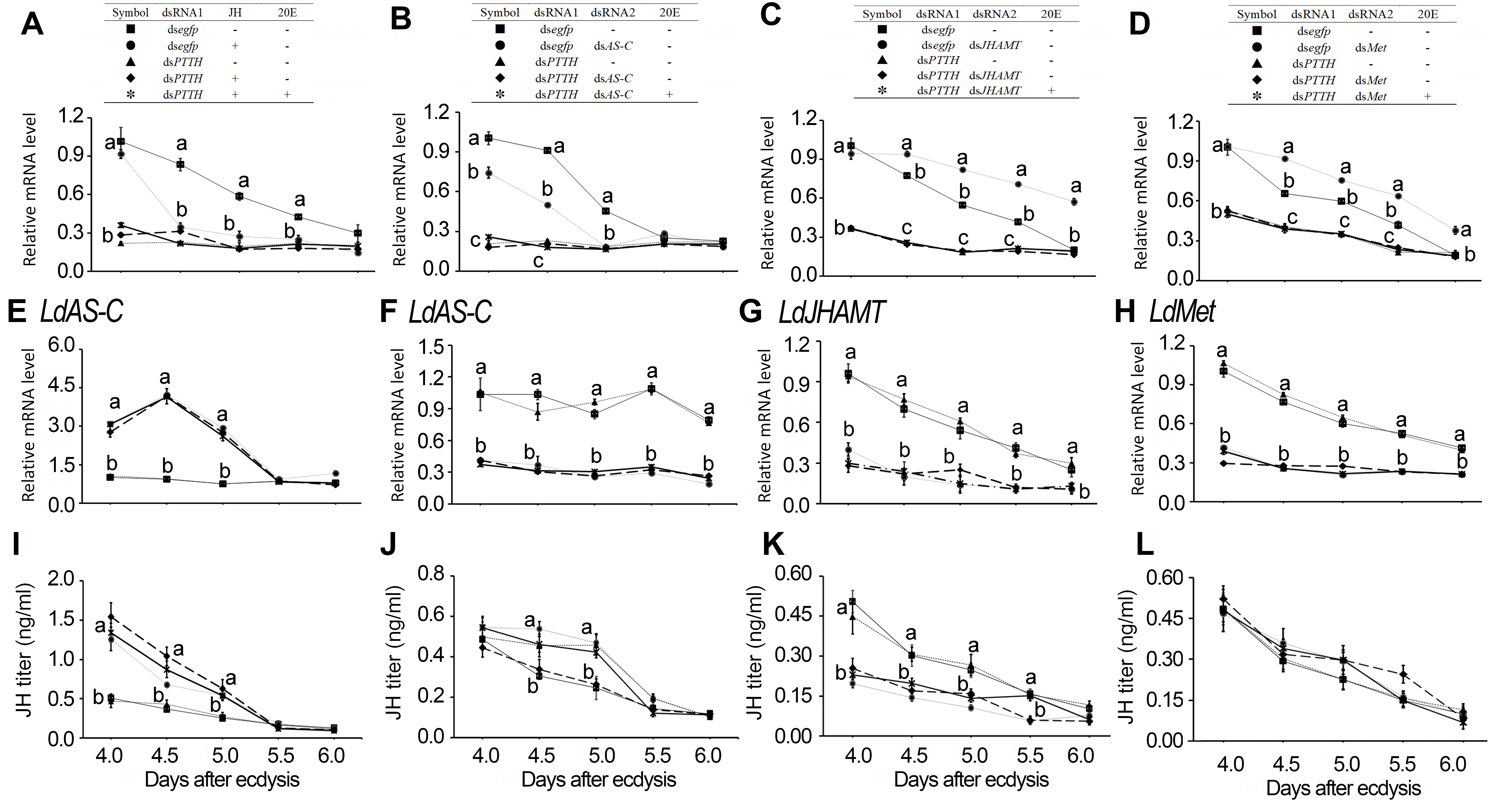

Supplement: S6 Fig — The larvae have fed on dsegfp, dsegfp+JH, dsPTTH, dsPTTH+JH and dsPTTH+JH+20E; dsegfp, dsegfp+dsAS-C, dsPTTH, dsPTTH+ dsAS-C and dsPTTH+dsAS-C+20E; dsegfp, dsegfp+dsJHAMT, dsPTTH, dsPTTH+dsJHAMT and dsPTTH+ dsJHAMT+20E; or dsegfp, dsegfp+dsMet, dsPTTH, dsPTTH+dsMet and dsPTTH+ dsMet+20E for three days. The expression levels of PTTH (A-D) and JH signal involved genes (E-H), and JH titers (I-L) were determined. (TIF) [file pgen.1007423.s006.tif]

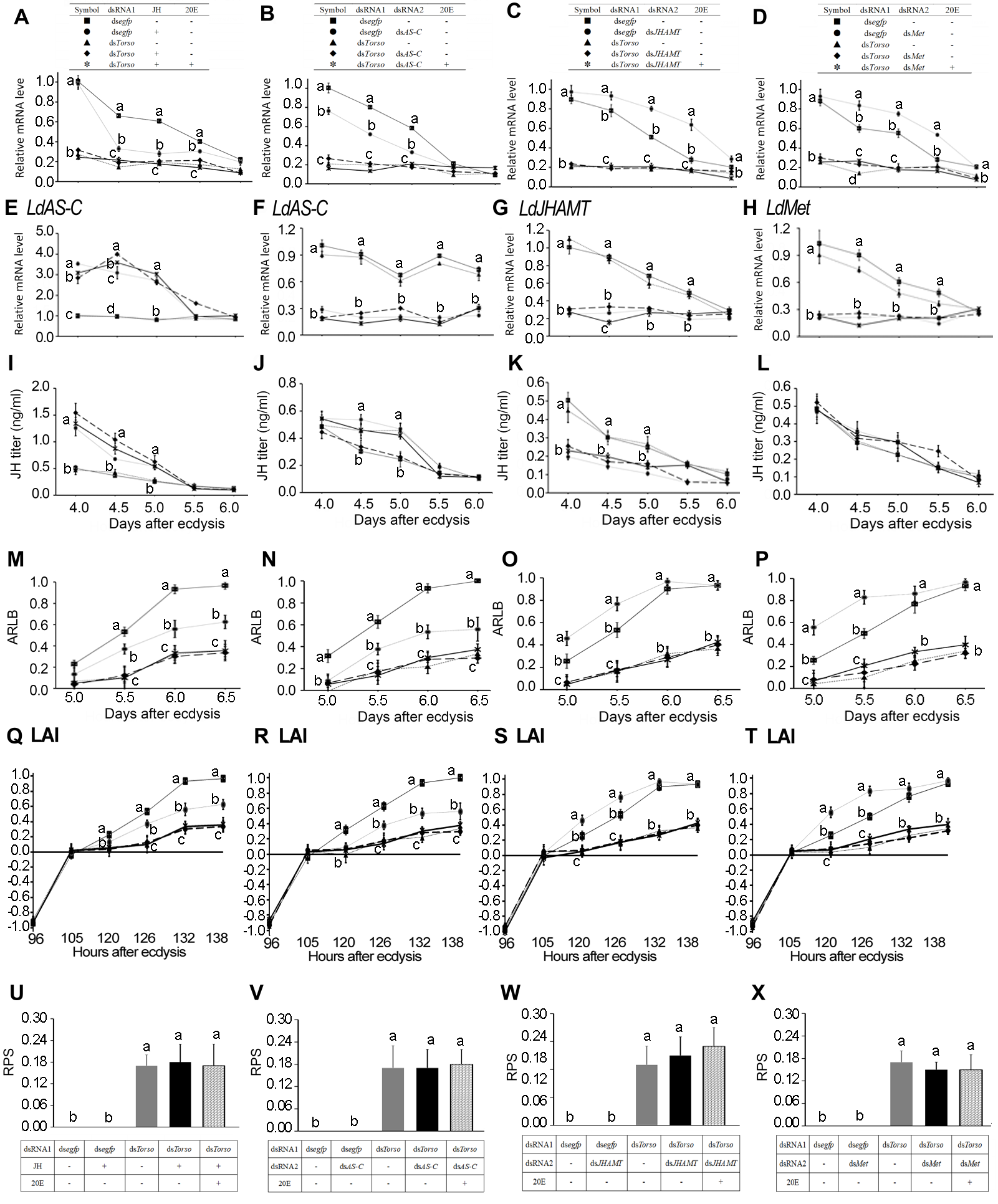

Supplement: S7 Fig — The expression levels of Torso (A-D) and JH signal involved genes (E-H), and JH titers (I-L) were disturbed by allowing the larvae to feed dsegfp, dsegfp+JH, dsTorso, dsTorso+JH and dsTorso+JH+20E; dsegfp, dsegfp+dsAS-C, dsTorso, dsTorso+ dsAS-C and dsTorso+ dsAS-C+20E; dsegfp, dsegfp+dsJHAMT, dsTorso, dsTorso+dsJHAMT and dsTorso+ dsJHAMT+20E; or dsegfp, dsegfp+dsMet, dsTorso, dsTorso+dsMet and dsTorso+ dsMet+20E. Significant differences in light avoidance index (LAI) (Q-T), accumulated rate of larvae that had buried in soil (ARLB) (M-P) at each testing time point, rate of pupae on the soil (RPS) (U-X) through a two-week experiment period to those in control (dsegfp-fed) were indicated by different letters (P < 0.05). (TIF) [file pgen.1007423.s007.tif]

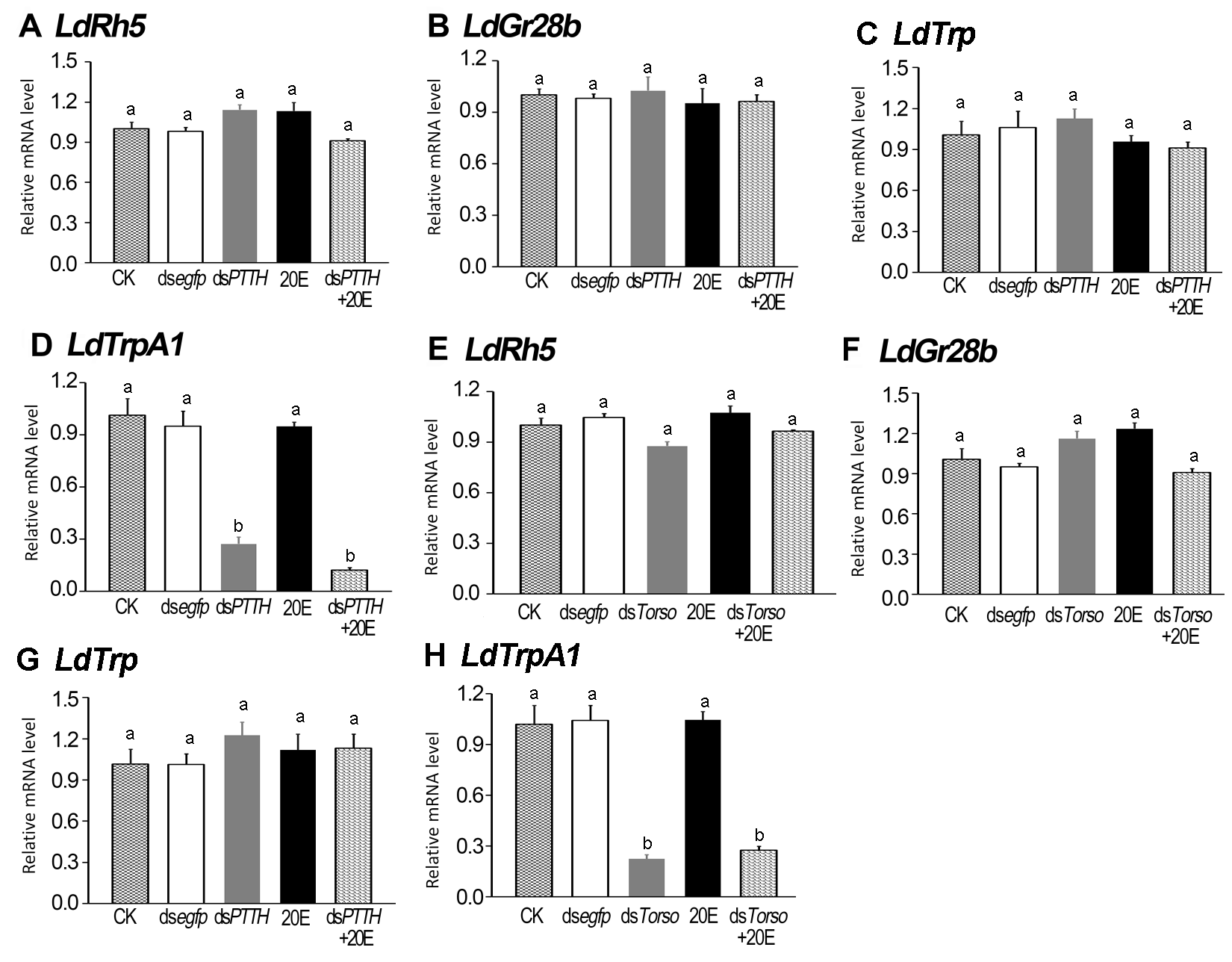

Supplement: S8 Fig — Newly-ecdysed Leptinotarsa third-instar larvae had fed on PBS-, dsegfp-, dsPTTH-, 20E-, or dsPTTH+20E, or PBS-, dsegfp-, dsTorso-, 20E-, or dsTorso+20E-immersed foliage for 3 days. Significant differences in the mRNA levels of four light sensing genes LdRh5, LdGr28b, LdTrp and LdTrpA1 were indicated by different letters (P<0.05). See legend in Fig 2 and S1 Fig. for further description. (TIF) [file pgen.1007423.s008.tif]

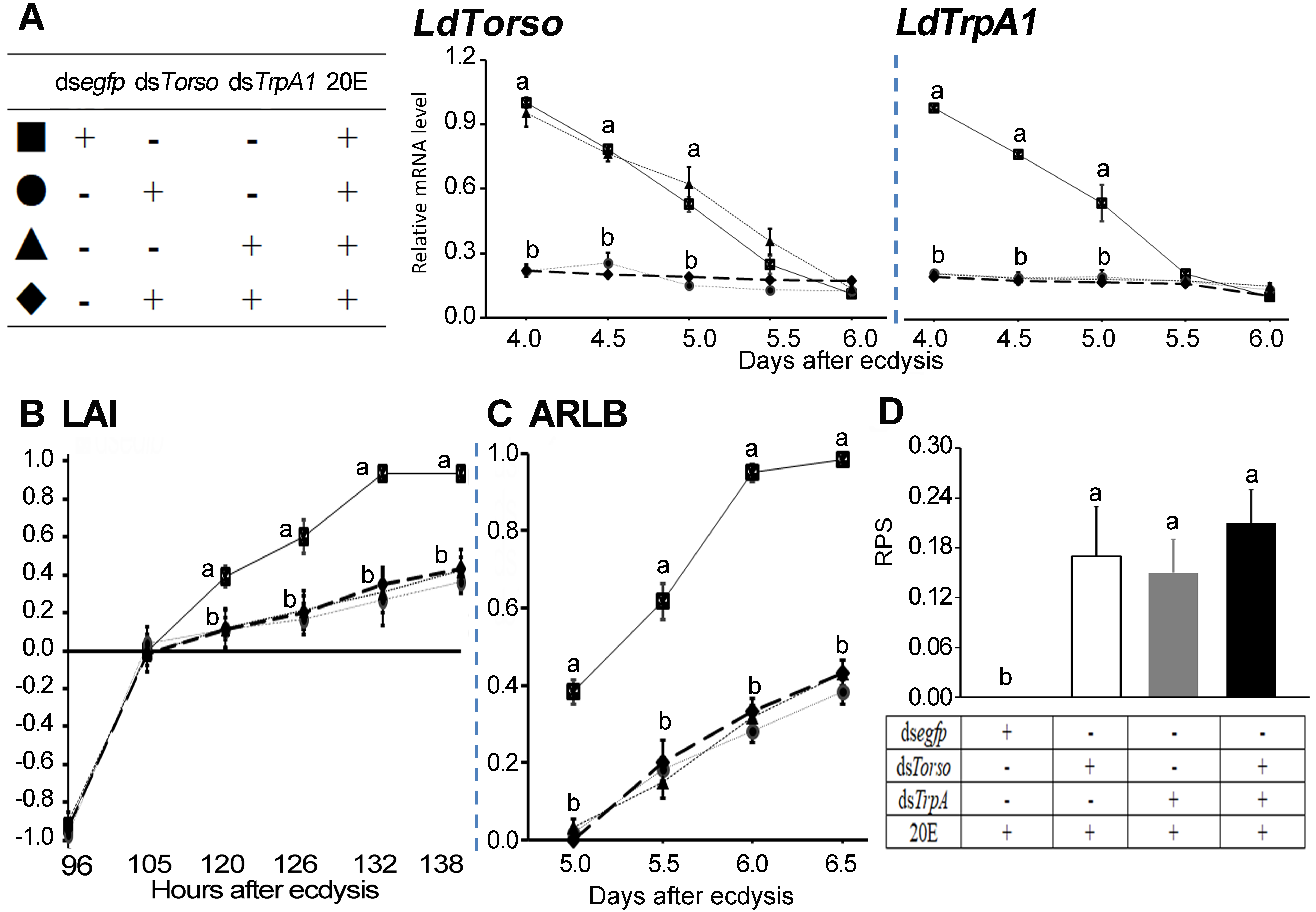

Supplement: S9 Fig — Newly-ecdysed Leptinotarsa third-instar larvae had fed PBS-, dsegfp-, dsTorso-, 20E-, or dsTorso+20E-immersed foliage for 3 days. Significant differences in transcript abundance of LdTorso and LdTrpA1 (A), light avoidance index (LAI) (B), accumulated rate of larvae that had buried in soil (ARLB) (C) at each testing time point, and rates of pupae on the soil (RPS) (D) through a two-week experiment period to those in control (dsegfp+20E-fed) were indicated by different letters (P<0.05). (TIF) [file pgen.1007423.s009.tif]

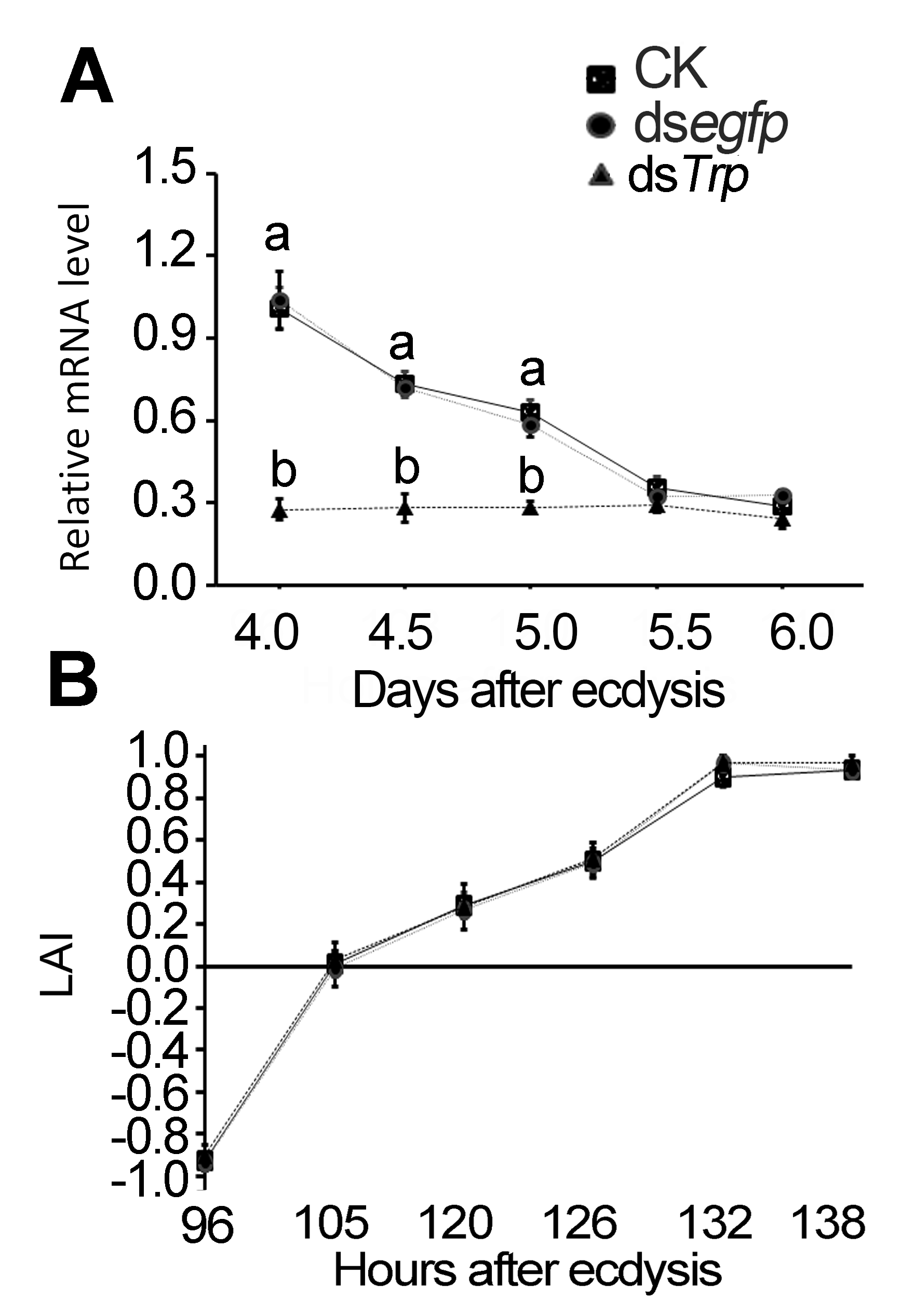

Supplement: S10 Fig — Newly-ecdysed Leptinotarsa third-instar larvae had fed PBS-, dsegfp- and dsTrp-immersed foliage for 3 days. The target gene was knocked down (A). No obvious differences in light avoidance index (LAI) (B) (P>0.10) were found in the LdTrp RNAi larvae. (TIF) [file pgen.1007423.s010.tif]
