# Supplementary material for: Hormonal signaling cascades required for phototaxis switch in wandering Leptinotarsa decemlineata larvae
Source: PLoS Genet. 2019 Jan 7;15(1):e1007423. doi: 10.1371/journal.pgen.1007423 (PMC6336328; doi:10.1371/journal.pgen.1007423)
Supplement: S1 Table — (PDF) [file pgen.1007423.s011.pdf]

Bioassay	Treatments	Days from ecdysis to the onset of wandering
**20E ingestion**	CK	4.7±0.4 b
	20E	4.0±0.3 a
**20E signal disturbance**	CK	4.5±0.2 a
	ds*egfp*	4.6±0.3 a
	ds*SHD*	5.3±0.2 b
	ds*EcR*	5.4±0.4 b
	ds*HR3*	5.2±0.3 b
**ILP2 knockdown**	CK	4.7±0.4 a
	ds*egfp*	4.5±0.2 a
	ds*ILP2*	5.4±0.3 b
**JH ingestion**	CK	4.6±0.2 a
	JH	5.5±0.4 b
**An increase in JH titer**	CK	4.7±0.4 a
	ds*egfp*	4.6±0.3 a
	ds*AS-C*	5.4±0.2 b
	ds*JHEH1*	5.1±0.2 ab
	ds*JHDK*	4.8±0.2 a
**JH signal interruption**	CK	4.7±0.2 b
	ds*egfp*	4.8±0.1 b
	ds*JHAMT*	4.0±0.2 a
	ds*Met*	4.1±0.3 a
**Knockdown of *LdPTTH***	CK	4.5±0.3 b
	ds*egfp*	4.6±0.2 b
	ds*PTTH*	5.8±0.3 a
	20E	3.7±0.4 c
	ds*PTTH*+20E	4.7±0.4 b
**Knockdown of *LdTorso***	CK	4.7±0.2 b
	ds*egfp*	4.5±0.4 b
	ds*Torso*	5.9±0.3 a
	20E	3.8±0.4 c
	ds*Torso*+20E	4.8±0.3 b
**A combination of *LdPTTH* knockdown and JH/20E application**	ds*egfp*	4.6±0.4 b
	ds*egfp*+JH	5.5±0.2 a
	ds*PTTH*	5.8±0.3 a
	ds*PTTH*+JH	5.9±0.2 a
	ds*PTTH*+JH+20E	4.7±0.3 b
**A combination of knockdown *LdPTTH* and *LdAS-C***	ds*egfp*	4.5±0.3 b
	ds*egfp*+ds*AS-C*	5.1±0.4 ab
	ds*PTTH*	5.9±0.3 a
	ds*PTTH*+ds*AS-C*	5.8±0.4 a
	ds*PTTH*+ds*AS-C*+20E	4.8±0.2 b
**A combination of knockdown *LdPTTH* and *LdJHAMT***	ds*egfp*	4.6±0.4 b
	ds*egfp*+ds*JHAMT*	4.1±0.3 c
	ds*PTTH*	5.7±0.3 a
	ds*PTTH*+ds*JHAMT*	5.8±0.2 a

	ds*PTTH*+ds*JHAMT*+20E	4.5±0.3 b
**A combination of knockdown *LdPTTH* and *LdMet***	ds*egfp*	4.6±0.3 b
	ds*egfp*+ds*Met*	3.9±0.2 c
	ds*Met*	5.9±0.3 a
	ds*PTTH*+ds*Met*	5.8±0.4 a
	ds*PTTH*+ds*Met*+20E	4.7±0.3 b
**A combination of *LdTorso* knockdown and JH/20E application**	ds*egfp*	4.6±0.3 b
	ds*egfp*+JH	5.8±0.4 a
	ds*Torso*	5.7±0.3 a
	ds*Torso*+JH	5.6±0.4 a
	ds*Torso*+JH+20E	4.6±0.2 b
**A combination of knockdown *LdTorso* and *LdAS-C***	ds*egfp*	4.5±0.3 b
	ds*egfp*+ds*AS-C*	5.9±0.2 a
	ds*Torso*	5.8±0.3 a
	ds*Torso*+ds*AS-C*	6.0±0.5 a
	ds*Torso*+ds*AS-C*+20E	4.8±0.2 b
**A combination of knockdown *LdTorso* and *LdJHAMT***	ds*egfp*	4.5±0.4 b
	ds*egfp*+ds*JHAMT*	3.8±0.2 c
	ds*Torso*	5.8±0.3 a
	ds*Torso*+ds*JHAMT*	5.6±0.3 a
	ds*Torso*+ds*JHAMT*+20E	4.6±0.4 b
**A combination of knockdown *LdTorso* and *LdMet***	ds*egfp*	4.7±0.3 b
	ds*egfp*+ds*Met*	4.0±0.2 c
	ds*Met*	5.7±0.3 a
	ds*Torso*+ds*Met*	5.8±0.3 a
	ds*Torso*+ds*Met*+20E	4.5±0.4 b
