# Supplementary material for: Hormonal signaling cascades required for phototaxis switch in wandering Leptinotarsa decemlineata larvae
Source: PLoS Genet. 2019 Jan 7;15(1):e1007423. doi: 10.1371/journal.pgen.1007423 (PMC6336328; doi:10.1371/journal.pgen.1007423)
Supplement: S2 Table — (PDF) [file pgen.1007423.s012.pdf]

Fragment name	Forward primer	Reverse primer
**dsRNA synthesis**	(bacterially)	
ds*SHD*	CTCTTCCTCGGTTATTCTTGCC	ATGCAAACCAGTTCAGGCC
ds*EcR*	ACTGCTCCGAGAAGACCAAAT	ATTTTCTCCACTTTCCAGCCT
ds*HR3*	GTGGAATAATGTAACGACCAAG	GAAGACGGCAGTACTGGC
ds*ILP2*	GTCCTCCTCGTCATCCTT	GTAACCTGTCGTGTCCCA
ds*AS-C*	GGCATCTGGGAGAAAATAG	GAAGTAGCAGGCTCTGAATCT
ds*JHEH1*	CTTCCTCATTATAGTCACA	CCTCAAGAGGTGGTGTTA
ds*JHDK*	ATCAGAGCGGCACCATAG	CTTCCCCTCAGCCATTTT
ds*JHAMT*	GGACAAGCCCGACTTATACTC	GCGAACTCCACCTCATCAA
ds*Met*	ACCTCTCCCGAAGACCACG	GCAACCTATTATGCGACCG
ds*PTTH*	TTGCTTCTAGTAACAACCCA	GAGTCTCAGATAATGGTCGG
ds*Torso*	CTAAATTCAAACCCCTTC	TCCCGTTACATTGCTACT
ds*TrpA1*	ACATTAATTCGACTTGGGGC	GTGTCAATCCACCACCATCA
ds*Trp*	TCATCCGCCCTAGTGATA	TTTGATTTGGGCTGAGAT
ds*NAT1*	TTTCATACCCAGAAGCTA	CCATGCTGTGTTTTCTGTCA
ds*egfp*	ACGTGACCACCCTGACCTA	GATGCCGTTCTTCTGCTTG
**dsRNA synthesis**	(lab-biosynthisizing)	
ds*Trp*	GGATCCTAATACGACTCACTATAGGGT CATCCGCCCTAGTGATA	GGATCCTAATACGACTCACTATAGGGT TTGATTTGGGCTGAGAT
ds*NAT1*	GGATCCTAATACGACTCACTATAGGGTT TCATACCCAGAAGCTA	GGATCCTAATACGACTCACTATAGGGC CATGCTGTGTTTTCTGTCA
ds*egfp*	GGATCCTAATACGACTCACTATAGGGA CGTGACCACCCTGACCTA	GGATCCTAATACGACTCACTATAGGGG ATGCCGTTCTTCTGCTTG
**qRT-PCR**		
q*LdSHD*	GGCCTGAACTGGTTTGCAT	GGCAAGAATAACCGAGGAAGAG
q*LdPHM*	GGAGAAAACTCGGAAAAGTGAAGA	CACCAAATAAATCAGCCAGCAA
q*LdEcR*	GAATGAGGGCAGAGTGTGTG	TCGTAGTGCTATTGGGCTTG
q*LdHR3*	GCATCATCCAGCAAATCATC	CTGATCGTCTTGCGACAACT
qLdFTZ-F1-1	GGCTAATCAGGCCTCCAG	CATGGTTTGCTGGCAACTAC

q*LdFTZ-F1-2*	ATGCATGAAGAAGCGACAAG	CTTCCAAATCCACTTCAGCA
q*LdILP2*	GTCCTCCTCGTCATCCTTGT	CCTGGGTTCTTGTCGTGTTA
q*LdInR*	TCTCAAGCGAATCAGAGGTG	GTTACGCCAATCCCAAAGTT
q*Ld4EBP*	TGCAGGGAGATCTCAACAAA	CAGAGAAGATCAACAAGCAGGA
q*LdAS-C*	ATGCAAGGCTTTCTCCATCT	TTGATTGTTGTCCGGGTCTA
q*LdKr-h1*	ATCAAGTGCCATTCCGAAAGCAA	ATGTCGGAATCATAGCTGACGGGT
q*LdJHEH1*	TGCAAGCTACGAAACCTGAC	TTGGTGCCAGATGTGAATTT
q*LdJHDK*	GTGTTACGGGCTGGATACCT	AGATGAAGTTACCGGGATCG
q*LdJHAMT*	GGAAGTGGAGATGGCAAGTT	CTACCAACGAGTTTCCCGAT
q*LdMet*	GCGACTCCGCTAACAGTGTA	AGATCCCTTCCTGGTTGTTG
q*LdPTTH*	TCCAAACTGGATCCTGTCAA	CACAGAGGAGGTTTCGTCAA
q*LdTorso*	CCAAAGTGCAGACTCCTCAA	CTTTGTTGCTCCCTCTTTCC
q*LdRas*	GACGTACAGGGAGCAGATCA	CTTGGCGACCTCTCTAGCTT
q*LdRh5*	TGGCTCGCCTGCCAGATTTA	TTCCCATCAAACGGCCTCGT
q*LdGr28b*	TGTTGTAACAGCTTACTTCACA	TAGGAAGTGGACATCGTCAT
q*LdTrpA1*	GGTGGTGGATTGACACCTCT	GTCCCTGTGCAACAAAGCTC
q*LdTrp*	TGACCGCATCTCAGCCCAAA	AGGAGCTGCATCGCAAAGGA
q*LdNAT1*	CCGGCACAGGCTTAGCATTCATTT	AGAGCTATCAGGCTTCCAACTCCT
q*LdRP18*	TAGAATCCTCAAAGCAGGTGGCGA	AGCTGGACCAAAGTGTTTCACTGC
q*LdRP4*	AAAGAAACGAGCATTGCCCTTCCG	TTGTCGCTGACACTGTAGGGTTGA
q*LdARF1*	CGGTGCTGGTAAAACGACAA	TGACCTCCCAAATCCCAAAC
q*LdARF4*	GTGCTCGTGAACCATGTGAA	AACCTCCAATCCCTCGTGAA
